# Impact of chromosomal polymorphisms on pregnancy outcomes after in vitro fertilization or intracytoplasmic sperm injection: A systematic review and meta-analysis

**DOI:** 10.12669/pjms.41.12.13037

**Published:** 2025-12

**Authors:** Liping Shen, Qiong Sun, Liwen Shen, Yurong Zhu, Yiwei Sun

**Affiliations:** 1 Liping Shen, Department of Emergency, Huzhou Maternity & Child Health Care Hospital, Huzhou, Zhejiang Province 313000, P.R. China; 2 Qiong Sun, Department of Emergency, Huzhou Maternity & Child Health Care Hospital, Huzhou, Zhejiang Province 313000, P.R. China; 3 Liwen Shen, Department of Centre for Reproductive Medicine, Huzhou Maternity & Child Health Care Hospital, Huzhou, Zhejiang Province 313000, P.R. China; 4 Yurong Zhu, Department of Centre for Reproductive Medicine, Huzhou Maternity & Child Health Care Hospital, Huzhou, Zhejiang Province 313000, P.R. China; 5 Yiwei Sun, Department of Emergency, Huzhou Maternity & Child Health Care Hospital, Huzhou, Zhejiang Province 313000, P.R. China

**Keywords:** Chromosomal Polymorphisms, Assisted Reproductive Technologies, Infertility, Spontaneous Abortion, Meta-Analysis

## Abstract

**Objective:**

Chromosomal polymorphisms (CPs), subtle variations in chromosome structure, have been previously reported in individuals with infertility, particularly among men. This systematic review and meta-analysis aim to analyze associations between CPs and pregnancy outcomes, focusing on spontaneous abortion rates.

**Methodology:**

A comprehensive literature search was conducted across three databases Medline, Google Scholar and Science Direct databases from inception to December 2023 for articles reporting the impact of CPs on selected fertilization and pregnancy outcomes among men/women/couples who had sought assisted reproductive technologies [ART; either *in vitro* fertilization (IVF) or intracytoplasmic sperm injection (ICSI)]. We applied the inverse variance method for pooled risk ratios (RR) with 95% confidence intervals (CIs).

**Results:**

From an initial 4271 articles, 16 studies met the inclusion criteria. The analysis evaluated CPs in relation to reproductive outcomes, including abortion rates (AR), fertilization rates (FR), cleavage rates (CR), clinical pregnancy rates (CPR), and good-quality embryo rates (GQER). No significant associations were observed between CPs and AR across men, women, or couples, though high heterogeneity suggested variability in study populations. CPs were associated with a modest reduction (7%) in FR (RR 0.93, 95% CI 0.88–0.97, I2 = 67.8; P-value = 0.04), and GQER (RR 0.93, 95% CI 0.89–0.96, I2 = 17.4; P-value = 0.30), and a small but statistically significant reduction CRs (RR 0.98, 95% CI 0.97–0.99, I2 = 0.0; P-value = 0.48).

**Conclusions:**

The study highlights the influence of CPs on certain fertilization and pregnancy outcomes, particularly GQER, FR and CR, while their impact on miscarriage and clinical pregnancy outcomes remains inconclusive. Further large-scale studies are necessary to investigate further associations.

## INTRODUCTION

In the realm of ART, especially in the context of IVF and intracytoplasmic sperm injections (ICSI), achieving successful pregnancies has remained a challenge.^1^ It is influenced by the interplay of numerous variables, such as maternal age, embryo quality, endometrial receptivity and underlying genetic abnormalities.^2^,^3^

CPs are heritable variations in the tightly packed heterochromatin segments of genomic DNA.^4^-^6^ These variants may include increased size or altered staining patterns of centromeric heterochromatin or structural rearrangements such as pericentric inversions, most frequently seen on chromosomes 1, 2 and 9.^7^ CPs carriers usually display normal function and phenotypes because heterochromatic DNA segments lack any protein-encoding function and are abundant in tandemly arranged, highly repetitive satellite DNA sequences.^8^ However, studies have shown that in couples undergoing ART procedures, the presence of CPs is associated with higher rates of failure, spontaneous abortion and unfavorable obstetric outcomes.^9^,^10^ Currently, the impact of CPs on IVF and/or ICSI outcomes remains unclear. Some studies have found no relevant effects, whereas others have found an association between CPs and selected pregnancy outcomes.^11^

The conflicting results may be due to study variations in methodologies, sample sizes and diagnostic criteria for chromosomal abnormalities, leading to a lack of consensus within the scientific community.^12^,^13^ This comprehensive meta-analysis aimed to consolidate the available data and provide a clear picture of the associations between CPs and assisted reproductive outcomes in couples with infertility

## METHODOLOGY

### Eligibility criteria and information sources:

The included studies reported on patients undergoing embryo transfer following either IVF or ICSI in an authorized health centre with data available for couples or for men/women separately, in which the study group participants had CPs and the control groups without CPs. Medline, Google Scholar and Science Direct databases were searched from inception to 31^st^ December 2023. The absence of similar systematic reviews on PROSPERO was confirmed and the protocol was registered [CRD42024499323]

The included studies focused on couples (or men and women separately) with CPs defined according to the International System for Chromosome Nomenclature.^14^ The CPs investigated included the pericentric heterochromatin length on the long arms of chromosomes 1, 9 and 16 (1qh ±, 9qh ± and 16qh ±); the short arm sizes (p +), satellites (PS +) and stalks (pstk +) of acrocentric chromosomes (chromosomes in D and G genomes); the distal chromosome Y heterochromatin (Yqh ±); and chromosome 9 and Y inversions.

### Search Strategy:

The following medical subject heading (MeSH) terms were used: “chromosomal polymorphism,” OR “karyotype polymorphism”, OR “chromosomal variant” AND “*in vitro* fertilization,” OR “assisted reproductive technology’, OR “intracytoplasmic sperm injection,” AND “pregnancy outcomes” OR “Fertilization outcomes” AND “Observational studies” OR “Cohort studies” OR “Prospective studies”. Table-II shows our detailed search strategy.

**Table-1:** Study summary.

Author year	Location	Identification of CP	Sample size	Design	Type of CP	Males: Females	Age (median and range/ Mean (SD))	Inclusion criteria	Outcomes	Quality of study (NOS)
Liang 2014	China	G-banding staining	614	Retrospective	Increase in distal heterochromatin of chromosome Y (Yqh+); an increase in the length of the pericentric hetero- chromatin on the long arms of chromosomes 1, 9 and 16 (1qh+, 9qh+ and 16qh+; respectively); double satellites on the short arm of chromosome 14 (14pss); variation in the length of the stalk on the short arm of chromosomes 13, 14, 15, 21, 22 (13pstk−, 14pstk±, 15pstk−, 21pstk+, 22pstk+, respectively); and pericentric inversion on chromosome 9	187:184	31.95 ± 6.84	infertile couples with CPs undergoing IVF-ICSI treatments in the Center of Reproductive Medicine	GQER, LBR, CPR, AR, BPR	7
Song 2017	China	Not provided	1108	Retrospective	Increase in distal heterochromatin of chromosome Y (Yqh+); an increase in the length of the pericentric hetero- chromatin on the long arms of chromosomes 1, 9 and 16 (1qh+, 9qh+ and 16qh+; respectively	Not available	Not available	Infertile couples, who received their first IVF/ICSI treatment cycles in health center	FR, CR, GQER, LBR	7
Xu 2016	China	G-band staining	847	Retrospective	qh+ (1, 9, 16 and Y chromosomes), qh− (Y chromosome), ps+ in D/G genomes and pericentric inversions (1, 9 and Y chromosomes)	348:99	68.3 ± 8.6	Infertile couples, who received their first IVF/ICSI treatment cycles in health center	FR, CR, GQER, LBR	8
Li 2016	China	G-band staining	20599	Retrospective	qh+ (1, 9, 16 and Y chromosomes), qh− (Y chromosome), ps+ in D/G genomes and pericentric inversions (1, 9 and Y chromosomes)	Not available	Not available	Infertile couples, who received their first IVF/ICSI	CR, GQER, LBR	8
Ni 2017	China	C-banding and R-banding staining methods	214	Retrospective	Not provided	125:86	30.86 ± 4.40	Infertile couples, who received their first IVF/ICSI	LBR, CPR, AR, OPR, BPR	7
Guo 2012	China	GTG-banded metaphase chromosomes (450-band resolutions)	281	Retrospective	Length of the pericentric heterochromatin on the long arms of chromosomes 1, 9 and 16 (1qh ±, 9qh ± and 16qh ±); the distal heterochromatin of chromo- some Y (Yqh ±); size of short arms (p +), satellites (ps +) and stalks (pstk +) of acrocentric chromosomes (chromosomes in D and G genomes); inversion of chromosomes 9 and Y	Not available	32.07 ± 4.36	Couples (<40 years) undergoing IVF or ICSI treatments	CPR, FR, OPR, AR, IR	8
Hong 2011	China	G-banding technique	1671	Retrospective	ength of the centromeric heterochromatin on the long arms of chromosomes 1, 9 and 16 (1qh+/2, 9qh+/2 and 16qh+/2) and the distal heterochromatic region of chromosome Y (Yqh+/2), distinct poly- morphic variants of the size of satellites (ps+) and lengths of stalks (pstk+) of the acrocentric (acro) chromosomes	187:82	Not available	Couples undergoing IVF or ICSI treatments	CPR, AR, OPR, IR	9
Li 2020	China	G-banding technique	1334	Retrospective	Polymorphic variations in centromeric heterochromatin length on the long arms of chromosomes 1, 9 and 16 (1qh, 9qh and 16qh)	131:115	30.88 ± 3.23	Infertile couples who had received their first IVF-embryo transfer treatment cycle and carried out karyo- type analyses	AR, CPR, IR	7
Liang 2023	China	G-banding technique under 450-band resolutions	1331	Retrospective	(i) qh+ group: 1qh+, 9qh+, or 16qh+; (ii) D/G group: 13 ph+, 14 ph+, 15 ph+, 21 ph+, 22 ph+, 22 ps+, 14pss, 22pss, 13pstk+, 14pstk+, 15pstk+, 21pstk+ and 22pstk+ (iii) inv(9) group: inv(9)(p12q13); (iv) Yqh+ group; and (v) Yqh group	187:89	Not available	Infertile couples undergoing IVF/ICSI treatment	CPR, AR, LBR, BPR	7
Ralapanawe 2022	Sri Lanka	G-banding technique	1879	Retrospective	Not available	200:150	34 ± 4.1	Couples undergoing karyotyping analysis followed by a cycle of ICSI treatment	LBR, AR, CPR	7
Rodriguez 2022	Spain	G-banding technique	1319	Retrospective	Long arm of chromosomes 1/9/16/Y were designated as qh+or qh-. An increase or decrease in lengths of the stalks on the short arm of the acrocentric chromosomes (D/G groups) was recorded as 13/14/15/21/22 pstk±. Double and increased satellites on the short arm on the same chromosomal group could also be observed and were designated as pss and ps+.	Not provided	Not provided	All couples under- went ICSI+PGT-A treatments	FR	6
Zhang 2023	China	G-banding technique	2740	Case control	Not provided	Not provided	31.88 ± 4.50	Couples with 40years undergoing IVF or ICSI used for fertilization	LBR, CPR, AR, BPR	7
Zhao 2023	China	G-banding technique	10400	Retrospective	Not provided	425:262	29.46 ± 4.13	Infertile couples undergoing fresh IVF/ ICSI-ET or FET treatments	CPR, AR, IR, LBR	7
Cao 2022	China	G-banding technique	585	Retrospective	Not provided	113:57	34.33 ± 4.61	Infertile couples undergoing fresh IVF/ ICSI-ET	CPR, AR, LBR	6
Liang 2019	China	G-banding technique	214	Retrospective	Couples with inv(9)(p12;q13)	53:54	32.37 ± 4.435	Infertile couples undergoing first IVF/ ICSI	CR, FR, IR, LBR, CPR, AR	7
Ciu 2022	China	G-banding technique	230	Retrospective	Not provided	64:49	Not provided	Infertile couples undergoing first IVF/ ICSI	AR, LBR	6

**Table-II:** Detailed search strategy.

SEARCH STRATEGY: PUBMED		
S.NO	SEARCH TERMS	RESULTS
#1	Search: **(chromosomal polymorphism) AND (in vitro fertilization)** (“chromosom”[All Fields] OR “chromosomally”[All Fields] OR “chromosome s”[All Fields] OR “chromosomes”[MeSH Terms] OR “chromosomes”[All Fields] OR “chromosomal”[All Fields] OR “chromosome”[All Fields] OR “chromosomic”[All Fields] OR “chromosomically”[All Fields] OR “chromosomics”[All Fields]) AND (“polymorphic”[All Fields] OR “polymorphics”[All Fields] OR “polymorphism s”[All Fields] OR “polymorphism, genetic”[MeSH Terms] OR (“polymorphism”[All Fields] AND “genetic”[All Fields]) OR “genetic polymorphism”[All Fields] OR “polymorphism”[All Fields] OR “polymorphisms”[All Fields]) AND (“in vitro fertilisation”[All Fields] OR “fertilization in vitro”[MeSH Terms] OR (“fertilization”[All Fields] AND “vitro”[All Fields]) OR “fertilization in vitro”[All Fields] OR (“vitro”[All Fields] AND “fertilization”[All Fields]) OR “in vitro fertilization”[All Fields]) **Translations** **chromosomal:** “chromosom”[All Fields] OR “chromosomally”[All Fields] OR “chromosome’s”[All Fields] OR “chromosomes”[MeSH Terms] OR “chromosomes”[All Fields] OR “chromosomal”[All Fields] OR “chromosome”[All Fields] OR “chromosomic”[All Fields] OR “chromosomically”[All Fields] OR “chromosomics”[All Fields] **polymorphism:** “polymorphic”[All Fields] OR “polymorphics”[All Fields] OR “polymorphism’s”[All Fields] OR “polymorphism, genetic”[MeSH Terms] OR (“polymorphism”[All Fields] AND “genetic”[All Fields]) OR “genetic polymorphism”[All Fields] OR “polymorphism”[All Fields] OR “polymorphisms”[All Fields] **in vitro fertilization:** “in vitro fertilisation”[All Fields] OR “fertilization in vitro”[MeSH Terms] OR (“fertilization”[All Fields] AND “vitro”[All Fields]) OR “fertilization in vitro”[All Fields] OR (“vitro”[All Fields] AND “fertilization”[All Fields]) OR “in vitro fertilization”[All Fields]	218
#2	Search: **((chromosomal polymorphism) AND (in vitro fertilization)) AND (ASSISTED REPRODUCTIVE TECHNOLOGY)** (“chromosom”[All Fields] OR “chromosomally”[All Fields] OR “chromosome s”[All Fields] OR “chromosomes”[MeSH Terms] OR “chromosomes”[All Fields] OR “chromosomal”[All Fields] OR “chromosome”[All Fields] OR “chromosomic”[All Fields] OR “chromosomically”[All Fields] OR “chromosomics”[All Fields]) AND (“polymorphic”[All Fields] OR “polymorphics”[All Fields] OR “polymorphism s”[All Fields] OR “polymorphism, genetic”[MeSH Terms] OR (“polymorphism”[All Fields] AND “genetic”[All Fields]) OR “genetic polymorphism”[All Fields] OR “polymorphism”[All Fields] OR “polymorphisms”[All Fields]) AND (“in vitro fertilisation”[All Fields] OR “fertilization in vitro”[MeSH Terms] OR (“fertilization”[All Fields] AND “vitro”[All Fields]) OR “fertilization in vitro”[All Fields] OR (“vitro”[All Fields] AND “fertilization”[All Fields]) OR “in vitro fertilization”[All Fields]) AND (“reproductive techniques, assisted”[MeSH Terms] OR (“reproductive”[All Fields] AND “techniques”[All Fields] AND “assisted”[All Fields]) OR “assisted reproductive techniques”[All Fields] OR (“assisted”[All Fields] AND “reproductive”[All Fields] AND “technology”[All Fields]) OR “assisted reproductive technology”[All Fields]) **Translations** **chromosomal:** “chromosom”[All Fields] OR “chromosomally”[All Fields] OR “chromosome’s”[All Fields] OR “chromosomes”[MeSH Terms] OR “chromosomes”[All Fields] OR “chromosomal”[All Fields] OR “chromosome”[All Fields] OR “chromosomic”[All Fields] OR “chromosomically”[All Fields] OR “chromosomics”[All Fields] **polymorphism:** “polymorphic”[All Fields] OR “polymorphics”[All Fields] OR “polymorphism’s”[All Fields] OR “polymorphism, genetic”[MeSH Terms] OR (“polymorphism”[All Fields] AND “genetic”[All Fields]) OR “genetic polymorphism”[All Fields] OR “polymorphism”[All Fields] OR “polymorphisms”[All Fields] **in vitro fertilization:** “in vitro fertilisation”[All Fields] OR “fertilization in vitro”[MeSH Terms] OR (“fertilization”[All Fields] AND “vitro”[All Fields]) OR “fertilization in vitro”[All Fields] OR (“vitro”[All Fields] AND “fertilization”[All Fields]) OR “in vitro fertilization”[All Fields] **ASSISTED REPRODUCTIVE TECHNOLOGY:** “reproductive techniques, assisted”[MeSH Terms] OR (“reproductive”[All Fields] AND “techniques”[All Fields] AND “assisted”[All Fields]) OR “assisted reproductive techniques”[All Fields] OR (“assisted”[All Fields] AND “reproductive”[All Fields] AND “technology”[All Fields]) OR “assisted reproductive technology”[All Fields]	165
#3	Search: **((chromosomal polymorphism) AND (in vitro fertilization)) AND (Pregnancy outcomes)** (“chromosom”[All Fields] OR “chromosomally”[All Fields] OR “chromosome s”[All Fields] OR “chromosomes”[MeSH Terms] OR “chromosomes”[All Fields] OR “chromosomal”[All Fields] OR “chromosome”[All Fields] OR “chromosomic”[All Fields] OR “chromosomically”[All Fields] OR “chromosomics”[All Fields]) AND (“polymorphic”[All Fields] OR “polymorphics”[All Fields] OR “polymorphism s”[All Fields] OR “polymorphism, genetic”[MeSH Terms] OR (“polymorphism”[All Fields] AND “genetic”[All Fields]) OR “genetic polymorphism”[All Fields] OR “polymorphism”[All Fields] OR “polymorphisms”[All Fields]) AND (“in vitro fertilisation”[All Fields] OR “fertilization in vitro”[MeSH Terms] OR (“fertilization”[All Fields] AND “vitro”[All Fields]) OR “fertilization in vitro”[All Fields] OR (“vitro”[All Fields] AND “fertilization”[All Fields]) OR “in vitro fertilization”[All Fields]) AND (“pregnancy outcome”[MeSH Terms] OR (“pregnancy”[All Fields] AND “outcome”[All Fields]) OR “pregnancy outcome”[All Fields] OR (“pregnancy”[All Fields] AND “outcomes”[All Fields]) OR “pregnancy outcomes”[All Fields]) **Translations** **chromosomal:** “chromosom”[All Fields] OR “chromosomally”[All Fields] OR “chromosome’s”[All Fields] OR “chromosomes”[MeSH Terms] OR “chromosomes”[All Fields] OR “chromosomal”[All Fields] OR “chromosome”[All Fields] OR “chromosomic”[All Fields] OR “chromosomically”[All Fields] OR “chromosomics”[All Fields] **polymorphism:** “polymorphic”[All Fields] OR “polymorphics”[All Fields] OR “polymorphism’s”[All Fields] OR “polymorphism, genetic”[MeSH Terms] OR (“polymorphism”[All Fields] AND “genetic”[All Fields]) OR “genetic polymorphism”[All Fields] OR “polymorphism”[All Fields] OR “polymorphisms”[All Fields] **in vitro fertilization:** “in vitro fertilisation”[All Fields] OR “fertilization in vitro”[MeSH Terms] OR (“fertilization”[All Fields] AND “vitro”[All Fields]) OR “fertilization in vitro”[All Fields] OR (“vitro”[All Fields] AND “fertilization”[All Fields]) OR “in vitro fertilization”[All Fields] **Pregnancy outcomes:** “pregnancy outcome”[MeSH Terms] OR (“pregnancy”[All Fields] AND “outcome”[All Fields]) OR “pregnancy outcome”[All Fields] OR (“pregnancy”[All Fields] AND “outcomes”[All Fields]) OR “pregnancy outcomes”[All Fields]	66
#4	**#1 AND #2 OR #3** ((“chromosom”[All Fields] OR “chromosomally”[All Fields] OR “chromosome s”[All Fields] OR “chromosomes”[MeSH Terms] OR “chromosomes”[All Fields] OR “chromosomal”[All Fields] OR “chromosome”[All Fields] OR “chromosomic”[All Fields] OR “chromosomically”[All Fields] OR “chromosomics”[All Fields]) AND (“polymorphic”[All Fields] OR “polymorphics”[All Fields] OR “polymorphism s”[All Fields] OR “polymorphism, genetic”[MeSH Terms] OR (“polymorphism”[All Fields] AND “genetic”[All Fields]) OR “genetic polymorphism”[All Fields] OR “polymorphism”[All Fields] OR “polymorphisms”[All Fields]) AND (“in vitro fertilisation”[All Fields] OR “fertilization in vitro”[MeSH Terms] OR (“fertilization”[All Fields] AND “vitro”[All Fields]) OR “fertilization in vitro”[All Fields] OR (“vitro”[All Fields] AND “fertilization”[All Fields]) OR “in vitro fertilization”[All Fields]) AND ((“chromosom”[All Fields] OR “chromosomally”[All Fields] OR “chromosome s”[All Fields] OR “chromosomes”[MeSH Terms] OR “chromosomes”[All Fields] OR “chromosomal”[All Fields] OR “chromosome”[All Fields] OR “chromosomic”[All Fields] OR “chromosomically”[All Fields] OR “chromosomics”[All Fields]) AND (“polymorphic”[All Fields] OR “polymorphics”[All Fields] OR “polymorphism s”[All Fields] OR “polymorphism, genetic”[MeSH Terms] OR (“polymorphism”[All Fields] AND “genetic”[All Fields]) OR “genetic polymorphism”[All Fields] OR “polymorphism”[All Fields] OR “polymorphisms”[All Fields]) AND (“in vitro fertilisation”[All Fields] OR “fertilization in vitro”[MeSH Terms] OR (“fertilization”[All Fields] AND “vitro”[All Fields]) OR “fertilization in vitro”[All Fields] OR (“vitro”[All Fields] AND “fertilization”[All Fields]) OR “in vitro fertilization”[All Fields]) AND (“reproductive techniques, assisted”[MeSH Terms] OR (“reproductive”[All Fields] AND “techniques”[All Fields] AND “assisted”[All Fields]) OR “assisted reproductive techniques”[All Fields] OR (“assisted”[All Fields] AND “reproductive”[All Fields] AND “technology”[All Fields]) OR “assisted reproductive technology”[All Fields]))) OR ((“chromosom”[All Fields] OR “chromosomally”[All Fields] OR “chromosome s”[All Fields] OR “chromosomes”[MeSH Terms] OR “chromosomes”[All Fields] OR “chromosomal”[All Fields] OR “chromosome”[All Fields] OR “chromosomic”[All Fields] OR “chromosomically”[All Fields] OR “chromosomics”[All Fields]) AND (“polymorphic”[All Fields] OR “polymorphics”[All Fields] OR “polymorphism s”[All Fields] OR “polymorphism, genetic”[MeSH Terms] OR (“polymorphism”[All Fields] AND “genetic”[All Fields]) OR “genetic polymorphism”[All Fields] OR “polymorphism”[All Fields] OR “polymorphisms”[All Fields]) AND (“in vitro fertilisation”[All Fields] OR “fertilization in vitro”[MeSH Terms] OR (“fertilization”[All Fields] AND “vitro”[All Fields]) OR “fertilization in vitro”[All Fields] OR (“vitro”[All Fields] AND “fertilization”[All Fields]) OR “in vitro fertilization”[All Fields]) AND (“pregnancy outcome”[MeSH Terms] OR (“pregnancy”[All Fields] AND “outcome”[All Fields]) OR “pregnancy outcome”[All Fields] OR (“pregnancy”[All Fields] AND “outcomes”[All Fields]) OR “pregnancy outcomes”[All Fields])) **Translations** **chromosomal:** “chromosom”[All Fields] OR “chromosomally”[All Fields] OR “chromosome’s”[All Fields] OR “chromosomes”[MeSH Terms] OR “chromosomes”[All Fields] OR “chromosomal”[All Fields] OR “chromosome”[All Fields] OR “chromosomic”[All Fields] OR “chromosomically”[All Fields] OR “chromosomics”[All Fields] **polymorphism:** “polymorphic”[All Fields] OR “polymorphics”[All Fields] OR “polymorphism’s”[All Fields] OR “polymorphism, genetic”[MeSH Terms] OR (“polymorphism”[All Fields] AND “genetic”[All Fields]) OR “genetic polymorphism”[All Fields] OR “polymorphism”[All Fields] OR “polymorphisms”[All Fields] **in vitro fertilization:** “in vitro fertilisation”[All Fields] OR “fertilization in vitro”[MeSH Terms] OR (“fertilization”[All Fields] AND “vitro”[All Fields]) OR “fertilization in vitro”[All Fields] OR (“vitro”[All Fields] AND “fertilization”[All Fields]) OR “in vitro fertilization”[All Fields] **chromosomal:** “chromosom”[All Fields] OR “chromosomally”[All Fields] OR “chromosome’s”[All Fields] OR “chromosomes”[MeSH Terms] OR “chromosomes”[All Fields] OR “chromosomal”[All Fields] OR “chromosome”[All Fields] OR “chromosomic”[All Fields] OR “chromosomically”[All Fields] OR “chromosomics”[All Fields] **polymorphism:** “polymorphic”[All Fields] OR “polymorphics”[All Fields] OR “polymorphism’s”[All Fields] OR “polymorphism, genetic”[MeSH Terms] OR (“polymorphism”[All Fields] AND “genetic”[All Fields]) OR “genetic polymorphism”[All Fields] OR “polymorphism”[All Fields] OR “polymorphisms”[All Fields] **in vitro fertilization:** “in vitro fertilisation”[All Fields] OR “fertilization in vitro”[MeSH Terms] OR (“fertilization”[All Fields] AND “vitro”[All Fields]) OR “fertilization in vitro”[All Fields] OR (“vitro”[All Fields] AND “fertilization”[All Fields]) OR “in vitro fertilization”[All Fields] **ASSISTED REPRODUCTIVE TECHNOLOGY:** “reproductive techniques, assisted”[MeSH Terms] OR (“reproductive”[All Fields] AND “techniques”[All Fields] AND “assisted”[All Fields]) OR “assisted reproductive techniques”[All Fields] OR (“assisted”[All Fields] AND “reproductive”[All Fields] AND “technology”[All Fields]) OR “assisted reproductive technology”[All Fields] **chromosomal:** “chromosom”[All Fields] OR “chromosomally”[All Fields] OR “chromosome’s”[All Fields] OR “chromosomes”[MeSH Terms] OR “chromosomes”[All Fields] OR “chromosomal”[All Fields] OR “chromosome”[All Fields] OR “chromosomic”[All Fields] OR “chromosomically”[All Fields] OR “chromosomics”[All Fields] **polymorphism:** “polymorphic”[All Fields] OR “polymorphics”[All Fields] OR “polymorphism’s”[All Fields] OR “polymorphism, genetic”[MeSH Terms] OR (“polymorphism”[All Fields] AND “genetic”[All Fields]) OR “genetic polymorphism”[All Fields] OR “polymorphism”[All Fields] OR “polymorphisms”[All Fields] **in vitro fertilization:** “in vitro fertilisation”[All Fields] OR “fertilization in vitro”[MeSH Terms] OR (“fertilization”[All Fields] AND “vitro”[All Fields]) OR “fertilization in vitro”[All Fields] OR (“vitro”[All Fields] AND “fertilization”[All Fields]) OR “in vitro fertilization”[All Fields] **Pregnancy outcomes:** “pregnancy outcome”[MeSH Terms] OR (“pregnancy”[All Fields] AND “outcome”[All Fields]) OR “pregnancy outcome”[All Fields] OR (“pregnancy”[All Fields] AND “outcomes”[All Fields]) OR “pregnancy outcomes”[All Fields]	173
***SCIENCE DIRECT***		
***S.NO***	***SEARCH TERMS***	***RESULTS***
#1	((chromosomal polymorphism) AND (in vitro fertilization)) AND (Pregnancy outcomes)	1955
***GOOGLE SCHOLAR:***		2143
	Chromosomal polymorphisms	
	Assisted reproductive technology	
	In vitro fertilization	
	Pregnancy outcomes	
	Fertilisation outcomes	
	Karyotype polymorphism	
	Chromosomal variant	
	Intracytoplasmic sperm injection	
	Cohort studies	
	Case-control studies	
	Cross-sectional studies	
	Observational studies	

### Search and data collection process:

The review followed the PRISMA framework.^15^ Two authors (LS and QS) independently conducted the primary and secondary publication screenings, and they resolved conflicts through mutual consensus, or by consultation with the third author (LS). Our review followed the PRISMA framework.^15^ During the primary screening, authors (LS and QS) assessed titles and abstracts, eliminating duplicates. During the secondary screening, they reviewed full texts considering the inclusion criteria, and they extracted all necessary information onto a pre-designed template sheet, double-checking the data for completeness and accuracy. The extracted data included author information, sample size, design, type of CP, inclusion criteria and study outcome.

### Data items:

The primary outcomes included 1) FR: the percentage of oocytes that become successfully fertilized after insemination via IVF or ICSI, 2) GQER: the proportion of embryos classified as morphologically high-quality (based on cell number, fragmentation, symmetry, etc.) at a defined stage, 3) CR: the proportion of fertilized oocytes (zygotes) that undergo the first mitotic division to form cleaved embryos, 4) early spontaneous AR: the proportion of clinical pregnancies that end in miscarriage before 12 weeks of gestation, 5) CPR: the proportion of cycles resulting in a pregnancy confirmed by ultrasound visualization of one or more gestational sacs.^16^ The included studies were of different analytical designs (cross-sectional, prospective and retrospective).

### Statistical Analysis:

Statistical analysis of all meta-analyses was performed using STATA software version 14.2. The pooled effect sizes were summarized as RR with 95% CIs using inverse variance method. A p-value < 0.05 was considered statistically significant. Heterogeneity across studies were quantified using the Q-test and the I2 statistic. Forest plots were generated to visualize the results using random effects model. Publication bias was not assessed due to the lack of adequate number of studies (at least 10) for any of the reported outcomes.

### Heterogeneity Assessment and quality of included studies:

Heterogeneity was assessed via *I^2^* and Chi-square tests and the studies were categorized as containing mild (*I^2^* < 25%), moderate (*I^2^* between 25% and 75%), or substantial (*I^2^* > 75%) heterogeneity. We applied the Newcastle Ottawa scale (NOS) (Lo et al., 2014) to assess study quality, considering outcomes ascertainment, study group selection, and comparability, allowing a maximum score of 9 for each study.

## RESULTS

**Fig.1:**
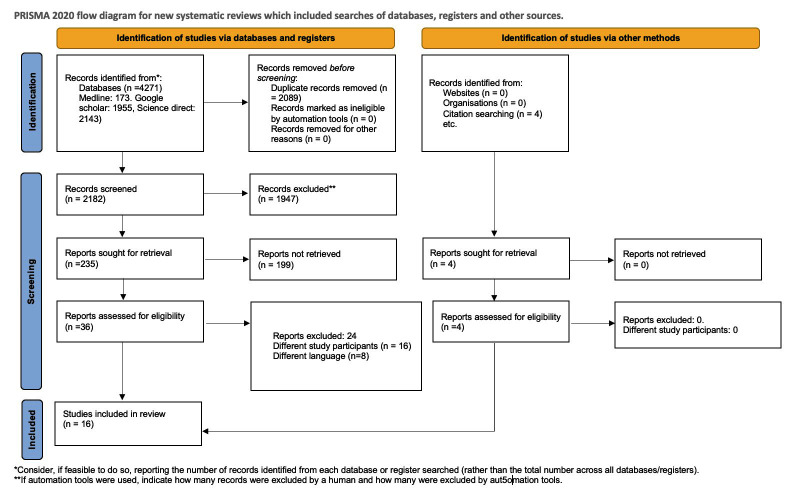
Flow diagram.

The initial search identified 4271 articles. After removing 2091 duplicated articles, a further 1947 articles were excluded during the title and abstract screening. Of the remaining 235 articles, 36 free full texts were retrieved for the secondary screening and four more articles were identified from citation searches. Out of the 36 full-text articles, 24 were excluded due to various reasons as mentioned. Finally, data from 16 articles (Fig.1) was analyzed.^7^,^8^,^11^,^13^,^17^-^27^ A total of 16 studies were included in the final review; the study characteristics are explained in Table-I. Eleven studies reported on AR, seven on CPR, six on FR, five on CR and four on GQER.

### CPs and AR:

**Fig.2:**
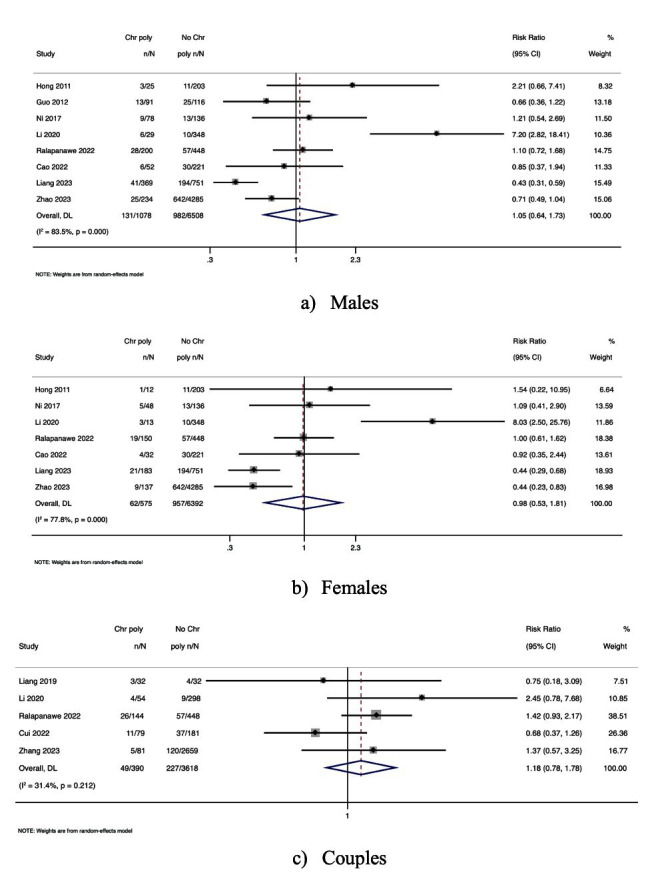
Forest plot of the association between CPs and AR: (a) Men; (b) Women; (c) Couples.

Eight studies reported the association between AR and semen samples from men with CPs [OR, 1.05; 95% CI, 0.64-1.73 with high heterogeneity, *I^2^* = 83.5; *P*-value <0.001] [Fig.2A]. Seven studies reported AR among women with CPs [OR, 0.98; 95% CI, 0.53-1.81 with high heterogeneity, *I^2^* = 77.7; *P*-value <0.001] [Fig.2B]. There was no association between couples with CPs and AR (3 studies) [OR, 1.18; 95% CI, 0.78-1.78 with nil heterogeneity, *I^2^ =* 31.4; *P*-value = 0.21] [Fig.2C]. Although no statistically significant associations were found, the high heterogeneity observed (I^2^ > 75%) in most comparisons suggests variability in study populations or diagnostic definitions.

### CPs and GOER:

**Fig.3:**
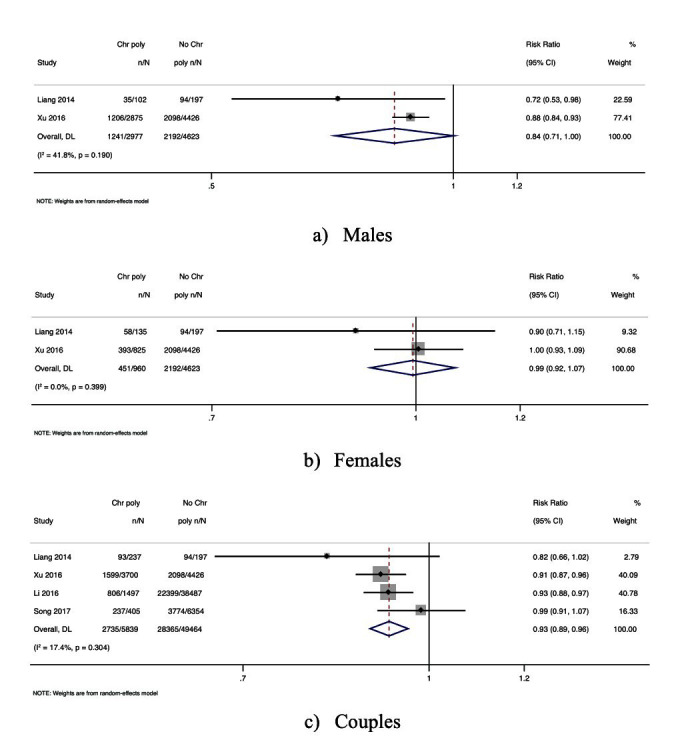
Forest plot of the association between CPs and the clinical pregnancy rates: (a) CPs in men; (b) CPs in women; (c) CPs in couples.

Two studies reported the association between GQER and semen samples from men with CPs [RR, 0.84; 95% CI, 0.71-1.00 with moderate heterogeneity, *I^2^* = 41.8; *P*-value = 0.19]. [Fig.3A] In addition, two studies assessed the association between GQERs and women with CPs [RR, 0.99; 95% CI, 0.92-1.07 with nil heterogeneity, *I^2^* = 0.0; *P*-value = 0.39]. [Fig.3B] However, there was a statistically significant association between couples with CPs and GQERs (four studies showing 7% reduction in GQER) indicating possible meiotic or epigenetic effects of CPs on embryo competence [RR, 0.93; 95% CI, 0.89-0.96 with low heterogeneity, *I^2^* = 17.4; *P*-value = 0.30] [Fig.3C].

### CPs and FR:

**Fig.4:**
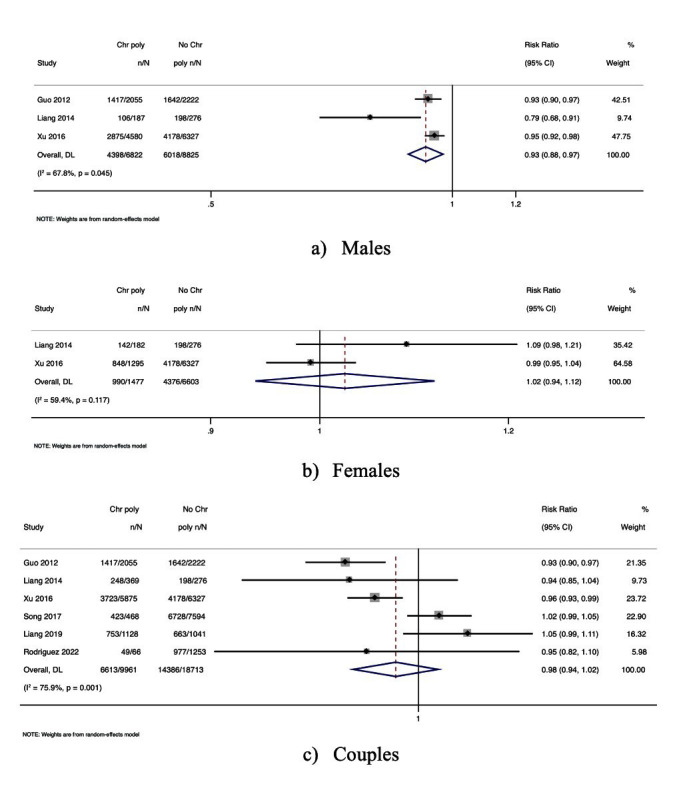
Forest plot of the association between CPs and fertilisation rate: (a) CPs in men; (b) CPs in women; (c) CPs in couples.

According to the pooled results, semen samples from men with CPs had 7% less risk of resulting in fertilisation than those from men without CPs [three studies, RR, 0.93; 95% CI, 0.88-0.97 with moderate heterogeneity, *I^2^* = 67.8; *P-* value = 0.04]. [Fig.4A] Two studies reported an association between FRs and women with CPs [RR, 1.02; 95% CI, 0.94-1.12 with moderate heterogeneity, *I^2^* = 59.4; *P*-value = 0.11] [Fig.4B] and 6 studies reported the association between couples with CPs and FR [RR, 0.98; 95% CI, 0.94-1.02 with moderate heterogeneity, *I^2^* = 75.2; *P*-value = 0.004] [Fig.4C]. This statistically significant result suggests that sub microscopic chromosomal rearrangements or chromatin instability may potentially affect sperm function.

### CPs and CPR:

**Fig.5:**
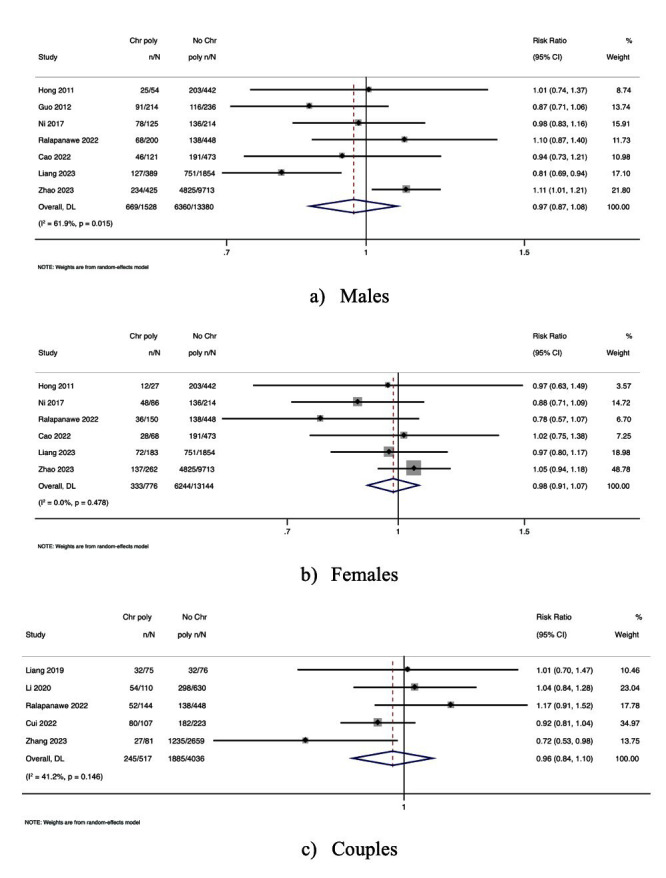
Forest plot explaining the association between CPs and the GOER: (a) CPs in men; (b) CPs in women; (c) CPs in couples.

Seven studies reported the association between CPR and semen samples from men with CPs [RR, 0.97; 95% CI, 0.87-1.08 with moderate heterogeneity, *I^2^* = 61.9; *P*-value = 0.01]. [Fig.5A] Six studies showed the association between CPR and women with CPs [RR, 0.98; 95% CI, 0.91-1.07 with nil heterogeneity, *I^2^* = 0.0; *P*-value = 0.47] [Fig.5B]. Five studies reported the association between CPR and couples with CPs [RR, 0.96; 95% CI, 0.84*-*1.10 with moderate heterogeneity, *I^2^* = 41.2; *P*-value = 0.14]. However, none of the above associations were statistically significant [Fig.5C].

### CPs and CR:

**Fig.6:**
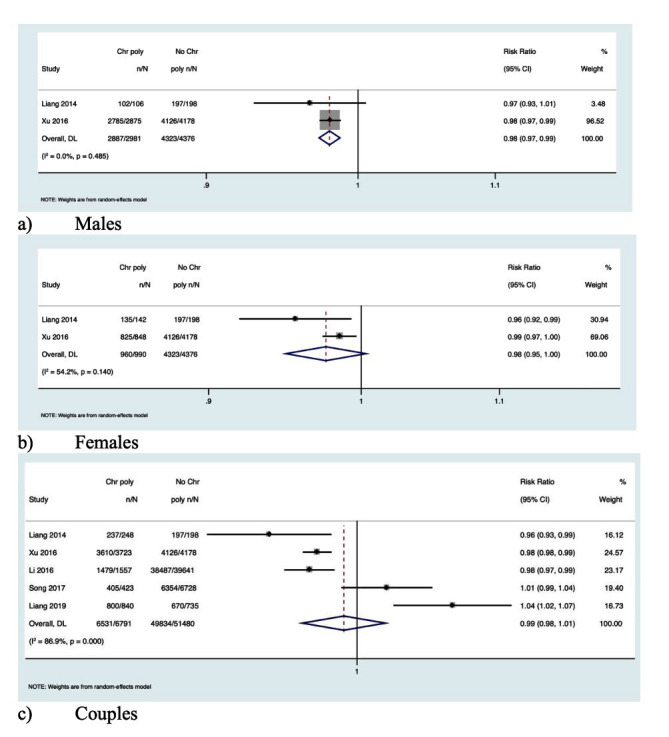
Forest plot explaining the association between CPs and the CR: (a) CPs in men; (b) CPs in women; (c) CPs in couples.

Two studies reported the association between CRs and women with CPs [RR, 0.98; 95% CI, 0.95-1.00 with moderate heterogeneity, *I^2^* = 54.2; *P*-value = 0.14] [Fig.6A]. However, there was a statistically significant association between CRs and semen samples from men with CPs (2% reduction in CR; RR, 0.98; 95% CI, 0.97-0.99 with moderate heterogeneity, *I^2^* = 0.0; P-value = 0.48) [Fig.6B], Fig.6 but not among couples with CPs (RR, 0.99; 95% CI, 0.98-1.01 with moderate heterogeneity, *I^2^* = 86.9; *P*-value = 0.009) [Fig.6C] The 2% reduction in cleavage rate in men with CPs (RR, 0.98) could indicate subtle paternal genomic effects during early embryogenesis, though the clinical relevance of this small reduction remains uncertain.

## DISCUSSION

The meta-analysis results from data in 16 studies estimated associations between CPs and IVF/ICSI outcomes. The study found that CPs reduced the GQERs and negatively impacted FRs and CRs (suggesting possible gametogenic or chromatin-level effects) after ART with samples from infertile men with CPs, but not in ART procedures in which only women had CPs. Low heterogeneity was detected for most associations, highlighting the low variability across the included studies. The findings on the association between CPs and several other outcomes, such as GQERs (nil effect), CRs (CPs in men resulted in lower CR than those in women) and FRs (CPs in men resulted in lower FR than those in women) are similar to those in a previous meta-analysis.^28^ Notably, this meta-analysis included more recent studies and assessed more pregnancy outcomes.

Our study results showed that semen samples from infertile men are often associated with adverse pregnancy outcomes. This may be due to several reasons. First, a main type of CPs altering sperm function is found on the heterochromatin of the long arm of the Y chromosome.^29^,^30^ According to the stratified study results by Gao *et al*, male infertility may be primarily caused by Y-associated CPs, resulting in Y chromosome microdeletion.^30^ Second, the heterochromatin associated with CPs may inhibit or even completely stop the expression of euchromatin genes.^31^ Third, given that the assembly of heterochromatin at the centromeres occurs during cell division, heterochromatin alterations may lead to meiotic abnormalities that hinder the development of viable spermatozoa.^32^ In addition, the impact of CPs on genetic material imbalances during gamete formation causes abnormal sperm chromatin structures, leading to DNA fragmentation and other related factors.^33^

### Implications:

This study further emphasizes that genetic counselling and discussions regarding potential preimplantation genetic testing (PGT) should be offered upon identification of structural rearrangements (such as a translocation, inversion, deletion, or insertion) in a parent. PGT has emerged as a viable option to identify inherited chromosomal configurations. Chromosomal testing, therefore, should be seen as a standard screening procedure for infertile couples undergoing ART procedures. Genetic counselling is strongly recommended for all carriers of chromosomal abnormalities.^34^ However, the existing evidence cautions against the premature universal adoption of PGT for aneuploidy because the technology is not without flaws: PGT for aneuploidy may inaccurately classify euploid embryos as aneuploid and the management of seemingly mosaic embryos remains uncertain. Some practitioners within the IVF field caution that PGT has yet to undergo comprehensive validation,^35^ suggesting that current analyses may be biased, influenced by clinics’ motivations to showcase innovation and capitalize on the associated financial incentives from offering ‘cutting-edge’ therapies. Thus, further research on the efficacy and implications of PGT for genetic testing is warranted.^36^

### Strength of the study:

The main strengths of this meta-analysis are a comprehensive search strategy, meticulous data screening and rigorous evaluation of study quality based on the Newcastle Ottawa Scale. The inclusion of studies with diverse sample sizes and geographical locations adds robustness to the findings. The study attempted to explore the association of different pregnancy and fertilisation outcomes with CPs while updating the previously available meta-analysis.

### Limitations:

The heterogeneity across studies, both in methodologies and participant characteristics, may have introduced biases. Additionally, the retrospective nature of most included studies rules out questions about causality. Moreover, not all the studies reported on every outcome of interest; a portion of the conclusions for certain subgroups were drawn from just two articles, which may have biased the analysis. Finally, most of the included studies were conducted in China. This geographic concentration may limit the generalizability of the findings to other populations, particularly given the potential for ethnic and genetic variability in CPs and their phenotypic effects. Although the search strategy was comprehensive and did not exclude studies based on geographic location, the final pool predominantly reflected Chinese cohorts, likely due to higher publication output in this field from that region. It is therefore important to interpret the findings with the understanding that they may largely reflect the genetic and clinical characteristics of East Asian populations.

## CONCLUSIONS

The current meta-analysis found negative correlations between ART procedures with semen from men with CPs and a subgroup of outcomes. These findings highlight the importance of educating patients with CPs about the possible risks associated with IVF and ICSI. In conclusion, this meta-analysis provides a nuanced perspective on the intricate association between CPs and IVF/ICSI outcomes. New studies should elucidate the complex genetic determinants of reproductive success in the context of assisted reproduction.

### Authors’ contributions:

**LS:** Literature search, study design and manuscript writing.

**QS, Liwen Shen, YZ and YS:** Data collection, data analysis and interpretation. Critical Review.

**LS:** Manuscript revision and validation and is responsible for the integrity of the study.

All authors have read and approved the final manuscript.
